# Mapping the Urban Lead Exposome: A Detailed Analysis of Soil Metal Concentrations at the Household Scale Using Citizen Science

**DOI:** 10.3390/ijerph15071531

**Published:** 2018-07-19

**Authors:** Gabriel M. Filippelli, Jessica Adamic, Deborah Nichols, John Shukle, Emeline Frix

**Affiliations:** 1 Department of Earth Sciences and Center for Urban Health, Indiana University–Purdue University Indianapolis (IUPUI), 723 W. Michigan St., Indianapolis, IN 46202, USA; jess.adamic@gmail.com (J.A.); deemorri@iupui.edu (D.N.); jshukle@iu.edu (J.S.); efrix@indiana.edu (E.F.); 2 Environmental Resilience Institute, Indiana University, Bloomington, IN 46202, USA

**Keywords:** lead poisoning, citizen-science, exposome, urban metals

## Abstract

An ambitious citizen science effort in the city of Indianapolis (IN, USA) led to the collection and analysis of a large number of samples at the property scale, facilitating the analysis of differences in soil metal concentrations as a function of property location (i.e., dripline, yard, and street) and location within the city. This effort indicated that dripline soils had substantially higher values of lead and zinc than other soil locations on a given property, and this pattern was heightened in properties nearer the urban core. Soil lead values typically exceeded the levels deemed safe for children’s play areas in the United States (<400 ppm), and almost always exceeded safe gardening guidelines (<200 ppm). As a whole, this study identified locations within properties and cities that exhibited the highest exposure risk to children, and also exhibited the power of citizen science to produce data at a spatial scale (i.e., within a property boundary), which is usually impossible to feasibly collect in a typical research study.

## 1. Introduction

Urban soils bear a lingering legacy of sometimes centuries of human occupation and industrialization (e.g., [1,2,3]). Among the many anthropogenic inputs to surface soils are metals, many of which, such as lead (Pb), have a very strong and permanent impact on human health and urban well-being. Indeed, the social, health, and economic cost of Pb exposure from urban soils is steep—far greater than the cost of targeted remediation of soil Pb hotspots [4,5,6,7,8,9,10,11]. The problem is that soil metal concentration are highly heterogeneous at the small scale because of multiple sources (house paint, automobile exhaust and debris, particulate matter from utilities, industrial sources, etc.), and barring the identification of particular emission sources and sinks, these metal hotspots prove exceedingly difficult to pinpoint [12,13,14,15]. Although new remote sensing techniques are promising as future geochemical mapping tools in cities [16], many obstacles to their effective deployment remain.

One of the sad realities when it comes to soil contamination, is that it is often identified not through soil measurements, but rather through human disease. For example, we wait until hundreds or even thousands of kids appear on public health records through high blood Pb levels before we can use this to map the urban Pb exposome [17,18,19], which is a harmful and backwards approach to protecting public health (e.g., [1,20]). What is needed is a better understanding of the soil metal dynamics and patterns of distribution and associations in the urban environment, with an eye toward identifying those processes and sources that have the highest potential to cause human harm, and ultimately remediating those sources in a surgical fashion [21,22,23,24,25].

**Figure 1:**
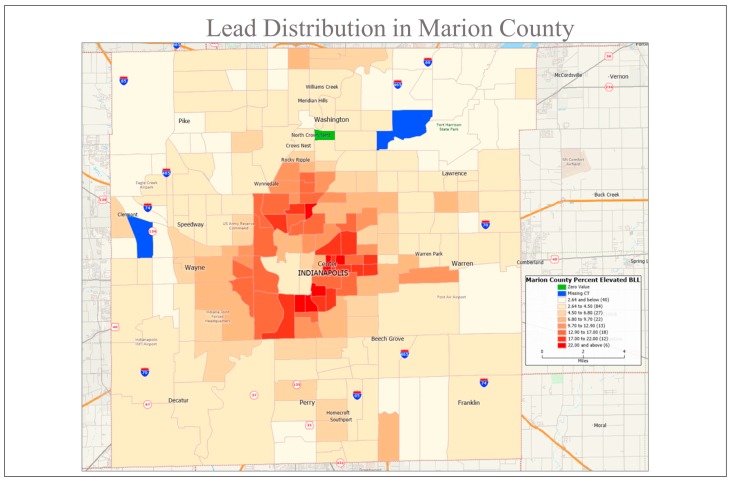
Map of blood lead levels (BLL) for children tested in Indianapolis from 2005–2015 aggregated at the census tract level (data from Reuter, 2018 [26]). These results reveal areas in the city with persistent Pb exposure to children, which is likely driven largely by soil and dust sources of legacy Pb from a mixture of leaded gasoline, lead-based paints, and industrial sources (Laidlaw and Filippelli, 2008 [23]).

We present results from one such effort to map the urban metal exposome in the city of Indianapolis, Indiana (USA), with a population of about one million people residing the in the heart of the post-industrial Midwest region. Indianapolis is a typical older (>150 years old) city with a substantial legacy of industrialization, manufacturing, and vehicle use, with urban and near-urban residential areas marked by 80–100 year-old single-family dwellings. Indianapolis shares another characteristic of many Midwest cities—high rates of blood Pb poisoning, with >20% of children below seven years old exhibiting blood lead levels above the level of concern (5 microgram/dL) in some of the central census tracts (Figure 1; data from 2005–2015; [26]). The majority of Pb exposure to these children comes from soil Pb, with the mechanism being inadvertent ingestion or inhalation of Pb-contaminated soils and/or dusts generated from soils [23]. Almost no current sources of Pb emissions are present in Indianapolis (as in most U.S. cities), nevertheless the strong footprint of legacy Pb is seen in surface soils, as the geochemical immobility of Pb and the past atmospheric deposition sources from degraded paint dust, automobile emissions, and industrial processes leave patterns that will persist for centuries without remediation [14]. The aim of this paper is to show how citizen science can be utilized to better constrain the geochemical fabric of human impacts on a typical city, in order to catalyze action in those areas where environmental quality is poor.

### Citizen Science in Action

**Figure 2:**
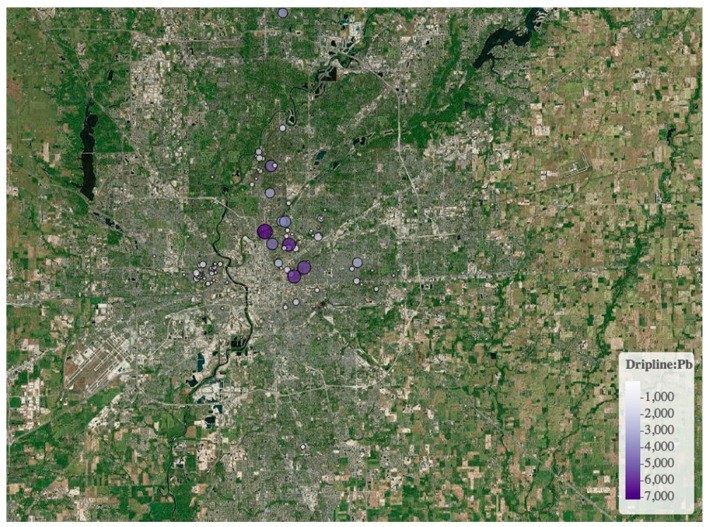
Example sample location map for Pb concentrations in dripline soils from Indianapolis, Indiana (USA), displayed as output from an application under development to assist citizen scientists in understanding the distribution patterns of metals in the city. Note that this represents roughly 500 individual properties, with multiple locations “hidden” under circles with high soil Pb concentrations.

A unique aspect of this study is that the majority of the samples were collected by “citizen scientists” under the Healthy Cities Project (http://kheprw.org/healthy-cities-project/). Citizen science is research conducted by amateur scientists, and is sometimes described as “public participation in scientific research” (e.g., [19]). In this project, the soil samples were guided by a soil Pb awareness and safety handbook, with instructions for sampling locations including a diagram of a typical home that defines the dripline (i.e., within one meter of the side of a home), the street side (within one meter of the edge of a road or sidewalk adjacent to a road), and the yard. We also provided instructions on how many samples to take (one dripline sample, one street side sample, and up to three other yard samples), sample collection (aggregate samples of approximately the top 10 cm), and sample storage (new zip-type plastic bags), in a manner similar as that employed in other settings (e.g., [17,27]). The geochemical analyses were performed at the Biogeochemistry and Health Core Facility at Indiana University-Purdue University Indianapolis (IUPUI), as described in the Methods, and the results and recommendations were returned to the citizen scientists. A total of just under 2000 samples were analyzed from approximately 500 residential properties (Figure 2), broadly covering the city. The program is ongoing, with a particular focus on youth activation and science communication.

We found clear patterns in soil metal concentrations and relationships between various metals at the household and the city level, providing insight into patterns of legacy metal deposition, patterns in human health risks, and potential sources of metals. The city-wide patterns for Pb in particular correlate strongly to blood lead levels of children, and the particularly high values found at house driplines provide further evidence that household-scale interventions that aim to isolate or cover near-home soils might go a long way toward reducing personal exposure risks for those dwelling in the homes.

## 2. Methods

A combination of open-call and campaign-style sampling schemes was utilized to collect soil samples from properties in Indianapolis. The open-call samples were solicited generally via on-line and in-person (community events, flyers, etc.) contacts, as well as promotion through the Marion County Public Health Department and Purdue Universities Agricultural Extension Office. The program began in 2012, and continues to be open to soil testing. The campaign-style samples came from individual communities or community groups who would canvas neighborhoods and take samples at high sampling densities—sometimes as frequently as every other property on a given block. This led to a total sample location pool that was neither random nor equally distributed around the city. We submitted ethics approval for this study through the Indiana University Institutional Review Board, and it was deemed exempt from human subjects reporting protocols, given that no human health or demographic data information was to be collected or published.

As noted above, instructions were provided as to the preferred location of samples, sampling depths, and sample labeling. However, with any citizen-generated science, the actual implementation of sampling was up to non-experts, and thus inevitably, some uncontrollable inconsistency is present in the sample pool. It is likely not biased in a specific direction, and furthermore, the sheer number of samples (~2000) is likely to effectively normalize some variations in sampling procedures, via the “normalization to the mean” nature of high sample n’s numbers. Some inconsistency in sample labeling was experienced, which could be controlled for. In some cases, this inconsistency was straightforward to interpret, such as “next to house” or “near road”, which translate into our pool of “dripline” and “street” categories. In some cases, however, samples were labeled in a manner that must have made sense to the citizen scientist (e.g., 1, 2, 3, 4, 5 or A, 2A, 3A, etc.), but could not be interpreted into functional categories and thus were excluded from this study. Finally, one key aspect of this analysis is the identification of spatial patterns in legacy metal concentrations, and in some cases, the sample address could not be uniquely geocoded to a specific location, and thus these samples were also excluded. Altogether, about 600 samples fell into these exclusion criteria, and thus the total number of samples presented here is about 1400.

Upon delivery to the laboratory, soils were processed and analyzed as per the literature [17]. Briefly, soil samples were dried, sieved to 150 microns, and weighed and ashed in a muffle furnace at 550 °C to degrade organic matter. The ashed sample was then transferred to 15-mL high density polyethylene disposable centrifuge tube and digested for two hours in 3N trace metal grade hydrochloric acid at 90 °C on a shaker table. After centrifugation, a subsample of the supernatant was diluted (1:100) with Milli-Q water and analyzed on a Perkin Elmer Inductively Coupled Plasma Optical Emission Spectrometer (ICP-OES) for a suite of metals, specifically lead (Pb), manganese (Mn), barium (Ba), chromium (Cr), copper (Cu), and zinc (Zn). Typical sample reproducibility, calculated from multiple measurements of the same soil ashed, digested, and analyzed, was 5% for all elements. Cadmium was also included in the initial analysis, but unacceptably high levels of detection and poor sample reproducibility precluded its effective use in this study.

## 3. Results

One of the critical components of predicting how, where, and to what metals people are exposed involves understanding both the origin of metal distribution patterns and the patterns of distribution at the personal (i.e., property) and community (i.e., neighborhood) scales. Our results for Indianapolis (Table S1) reveal two origin classes of metals found in surface soils, an anthropogenic source that dominate Pb and Zn distribution, and an ambient soil mineral source that defines the relatively homogenous distribution at the household and neighborhood scale of the other studied metals. The results confirm existing paradigms that elevated soil Pb in cities clearly have an anthropogenic origin and are generally highest near home driplines. However, the results also reveal the power of citizen science to take an active role in understanding the urban Pb exposome and to help fill the gap in the currently poor state of urban soil geochemical mapping.

### 3.1. Property-Scale Distribution of Soil Metals and Metal Sources

**Figure 3:**
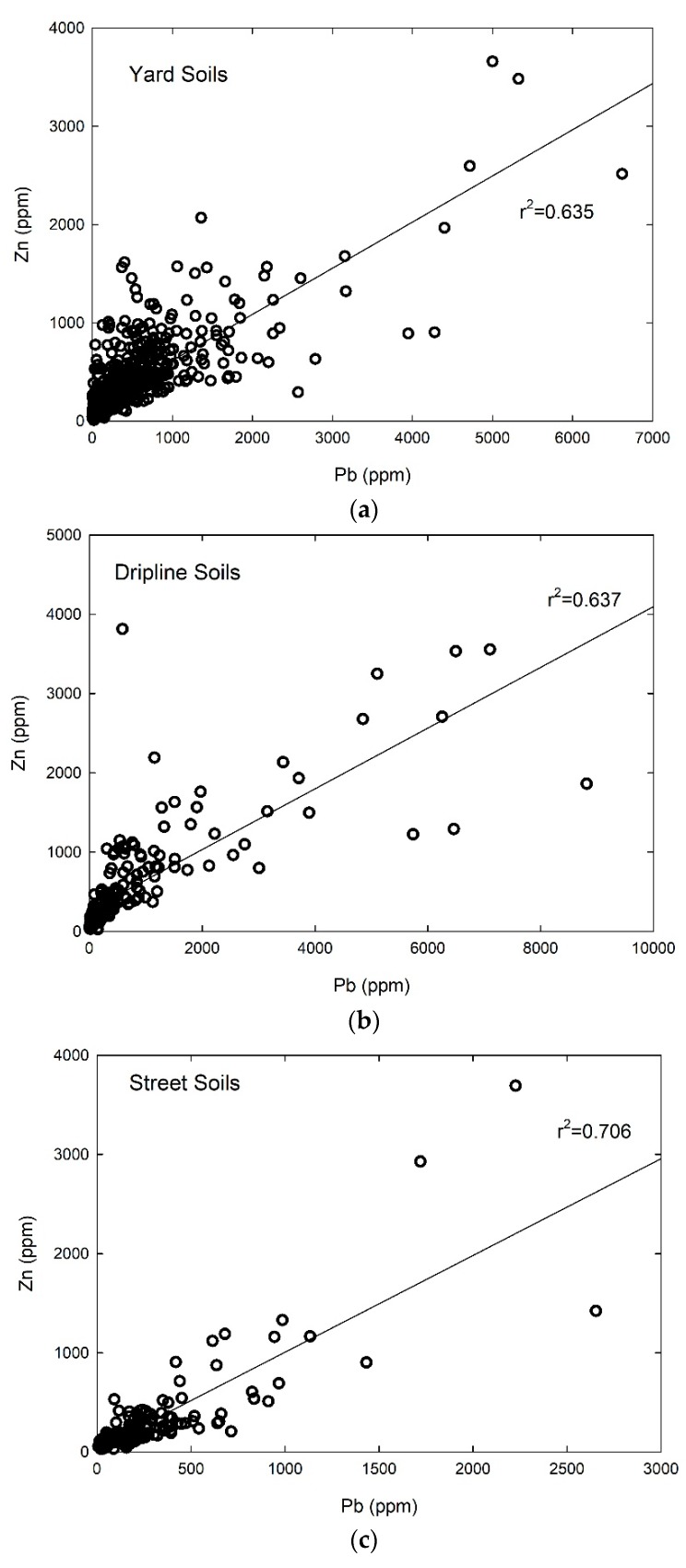
Correlation plots of lead (Pb) and zinc (Zn) for all soil location types. (**a**) Yard soil, (**b**) dripline soil, and (**c**) street soil. The similar slopes for each setting, the strong correlation between them, and their relative enrichment over average soil indicate a strong anthropogenic source impacting their distribution in urban soils.

Lead and Zinc. The greatest predictor of soil Pb and Zn concentration is location on a particular property. For both metals, concentrations are much greater at the dripline than in a given yard or near the street. For Pb, this results in mean concentrations near the home driplines of 805 parts per million (ppm, equivalent to mg/kg), with maximum values as high as 8816 ppm, and for Zn, the mean is 575 ppm and the maximum is 3814 ppm (Table 1). For Pb, mean yard samples are lower (345 ppm) and street samples even lower (240 ppm) than driplines, resulting in a relative dripline enrichment of 2.33. The enrichment factor is defined as the ratio of the highest to the least mean value in a given property location category (i.e., dripline, yard, street). For most elements, this is a ratio of dripline to yard values. For Zn, mean yard samples are also lower (312 ppm) and street samples even lower (262 ppm) than driplines, resulting in a relative dripline enrichment of 2.19, quite similar to that for Pb. This property-scale distribution pattern is an indicator of anthropogenic source(s) for these metals. Indeed, a strong correlation is observed between Pb and Zn when considering the entire individual sample population by location category, with r^2^ ranging from 0.637 for dripline samples, 0.635 for yard samples, and 0.706 for street samples (Figure 3). The consistency in correlation between these two elements across location category (and nearly identical slope; Figure 3) suggests a similar source for both metals. This was confirmed by a Principle Component Analysis (PCA) analysis of a subset of the soil samples analyzed by X-ray diffraction (XRF) (Figure S2), which revealed that Pb and Zn clustered strongly as a secondary factor (PCA2), whereas a host of other elements clustered in the more dominant PCA1, which correlated with geogenic mineralogies.

Perhaps the best baseline with which to reference urban soil metal concentrations is the comprehensive nation-wide United States Geological Survey (USGS) analysis [28]. This baseline encompasses rural regions that have no clear anthropogenic sources and other semi-urban and urban regions with a strong imprint of anthropogenic inputs. This U.S. average was chosen as it spans multiple soil types and mineralogical compositions. It is heavily biased toward non-urban settings given that the sample locations were based on a geographic grid that provides a “normal” spatial sample distribution, whereas urban and industrial centers are anything but normally distributed, with strong concentration and non-random distributions in the spatial sense.

Other Metals. Unlike the case for Pb and Zn, there are no apparent location patterns, nor substantial enrichments, for soil Mn, Ba, Cr, or Cu (Table 1). Driplines tend to have slightly higher Ba, Cr, and Cu values, but only by about 15–46%. The strongest dripline signature is for Cu (46% enrichment), which might support a weak relationship with an anthropogenic source. Meanwhile, Mn shows no enrichment at all at the property scale, which indicates a geogenic driver for soil Mn concentrations, even in an urban environment.

### 3.2. City-Scale Distribution of Soil Metals

There were typically three yard samples taken per property, as opposed to one each for dripline and street samples. Thus, the most statistically significant measure of city-wide patterns in soil metals lies in an analysis of yard soil samples. This analysis yields mean values for soil Pb of between 263 and 494 ppm for center township properties (i.e., downtown or near downtown), and between 323 and 468 ppm Zn (Table 2). Soil values were significantly lower outside of the downtown, with mean values between 157 and 175, respectively, for Pb and 239 and 244, respectively, for Zn (Table 2). It is important to note that fewer samples were taken outside of the downtown, and thus only two zipcodes had adequate (i.e., more than 20) samples from which to determine a mean value. No clear downtown to outside of downtown patterns were observed for the other elements analyzed as part of this study.

## 4. Discussion

### 4.1. Anthropogenic Sources for Pb and Zn

As has been amply documented, higher-than-background Pb and Zn values are consistent with an anthropogenic footprint in cities (e.g., [29,30]). The sources of these metals are multiple, and include leaded gasoline; lead-based paint, and industrial emissions for Pb and tire debris; fossil fuel combustion; and industrial sources for Zn [21]. This urban effect for Pb and Zn is even seen clearly in river and reservoirs sediment samples downstream of major metropolises [31] and is interpreted to derive from soil and dust runoff. Urban sources drive strong correlation in downstream sediments and reservoirs surface samples.

The soil values that we found for all sample types (dripline, yard, and street) for Pb and Zn were significantly above the U.S. mean [28]. For Pb, that enrichment was over 1000% for all sample types, and for Zn was over 400% for all sample types (Table 1). Although the strong correlation between Pb and Zn (Figure 3) and the similar household-scale enrichment factor (Table 1) suggest a similar source for both in the urban setting, the increase above mean U.S. soil values is much higher for Pb, with Pb being roughly 4.5 times higher than Zn in dripline soils, 3.6 in yard soils, and 2.9 in street soils. Collectively, this trend points to a house-based proximal source for Pb that is greater than that for Zn. Although a likely culprit might be lead-based paints, it is difficult to rule out the potential impact of past combustion of leaded gasoline and barrier capture of that aerolized Pb next to structures.

Both Pb and Zn were much higher in downtown properties than in those outside of the downtown. One interpretation of this pattern is the growth trajectory of Indianapolis, and indeed that of many Midwestern industrial cities. Densely spaced residential and light industrial land uses were common in the first half of the 20th century in downtown areas, and this co-location of emissions sources with high anthropogenic footprints likely lead to significantly higher amounts of industrial and vehicular emission and deposition of Pb and Zn, which retained their urban footprint [17,29] long after the phase-out of leaded gasoline, lead-based paints, and emission controls for small-scale Pb foundry and recycling facilities in Indianapolis [21]. Subsequent growth, in a relatively Pb-free time, occurred outside of the downtown area, and thus the anthropogenic legacies were significantly lower in these areas.

### 4.2. Implications for Human Health

One of the clearest outcomes of this research is that soils near homes pose significantly greater contact risks of Pb to individuals than soils anywhere else on their property. Indeed, mean dripline soil concentrations (805 ppm) are above the screening level of 400 ppm for soil Pb in playgrounds and children’s play areas [32], and those for mean yard soils (345 ppm) are barely below this cutoff. The consistent finding of high values at driplines is critical, as this is a source of soil and dust tracked into homes [33,34]. Further, the mean for all soil location types exceeds the safe gardening recommendations of 200 ppm Pb recommended by some researchers (e.g., [35]), and would benefit from interventions to ensure that gardening in not a undue source of Pb exposure either through direct contact or through produce consumption [36,37,38]. If one takes the more conservative, risk-based screening level of 100 ppm adopted by European countries [39], nearly all of the dripline and most of the full soil sample set would be in violation of standards.

To be sure, significant property-to-property variability exists, and beyond some city-scale patterns, it is impossible to predict in aggregate if a particular dripline soil Pb value will be 10 ppm (the minimum found in Indianapolis; Table S1) or 8816 ppm, the maximum found, (Table 1) and a level that clearly poses dangers to residents within and near to that home. Given the observed variability in dripline soils, and even those from yards and streets, it is fruitless to use mean values to predict Pb exposures to children based on a typical Integrated Exposure Uptake Biokinetic IEUBK soil exposure model. Indeed, even when property data was determined in those areas where denser sample coverage was achieved, such as on the near west side of Indianapolis, the property-to-property variability typically exceeded 50% across all sample types. Note that the data were aggregated at the zipcode level in this analysis to protect anonymity of individual property owners, and thus the source data for the block-scale analysis are not publicly available.

### 4.3. City-Wide Patterns in Anthropogenic Metal Distributions

Not surprisingly, downtown soils retained a much high legacy metal impact than those outside of the downtown. This indicates that risks from metal exposure are also significantly higher near the downtown core of Indianapolis, and likely many other similar cities in the American Midwest and east coast with similar histories and growth trajectories. Basically, when a city developed and its growth patterns dictate its anthropogenic metal footprint, with younger cities largely avoiding the worst of harmful metal emissions and legacy metal contaminants because of much more rigorous environmental protections that were implemented over the latter part of the 20th century in the United States.

## 5. Conclusions

The anthropogenic footprint or urbanization is easily observed in legacy metals distribution in Indianapolis, with the older urban core generally exhibiting the highest values of those metals presumed to be most strongly related to human activities—Pb and Zn. Beyond this urban concentration is another spatial pattern—that is, significantly elevated levels of Pb and Zn near home driplines. This indicates the role of structures in capturing fugitive dust, as well as being point sources of metals via paint and other building products. From a health protection standpoint, these findings reveal that urban core properties hold the greatest Pb exposure risk, and that soils proximal to structures are particularly concerning and warrant special attention to either remove the source or encapsulate it via robust groundcover. An interesting outcome of the approach to sample collection is that citizen-scientists can be effectively engaged to expand research and to provide data at a scale that is both not feasibly collected by an individual research and that are most characteristics of the household’s individual soil exposure risk situation. Future work focusing on the efficacy of risk communication and more spatially-explicit examination of soil Pb values versus blood lead levels at the neighborhood scale would further increase the public health impact of citizen science studies such as this one.

## Figures and Tables

**Table 1:** Metals concentrations (ppm) for soils as a function of location on a property. Enrichment factor is calculated as highest value/lowest value for each mean metal concentration. Lead (Pb), manganese (Mn), barium (Ba), chromium (Cr), copper (Cu), and zinc (Zn).

Location of Samples	Drip	Yard	Street	Enrichment	U.S. Mean *
	187	976	192		(ppm)
Pb					
Mean	805	345	240	2.33	18
Median	282	193	143		
Max	8816	6619	2654		
Mn					
Mean	635	695	646	0.98	492
Median	603	666	616		
Max	1359	2597	1358		
Ba					
Mean	147	132	118	1.25	510
Median	124	116	104		
Max	1100	1200	969		
Cr					
Mean	23	22	20	1.15	30
Median	20	19	19		
Max	249	1183	79		
Cu					
Mean	41	28	29	1.46	14
Median	21	18	16		
Max	1469	1332	888		
Zn					
Mean	575	312	262	2.19	58
Median	313	216	149		
Max	3814	3660	3892		

* United States Geological Survey (USGS) soil geochemical survey of conterminous US 0–5 cm [28].

**Table 2:** Concentration of yard soil Pb and Zn sorted by Zipcode (only where over 20 samples were tested). Indianapolis, Indiana. Center township represents the downtown and near downtown zipcodes.

Zipcode	Value	Pb	Zn
		ppm	ppm
Center Township			
46218	mean	373	323
	median	216	236
46222	mean	471	374
	median	257	292
46201	mean	401	340
	median	261	246
46202	mean	666	468
	median	376	293
46203	mean	495	421
	median	279	289
46208	mean	263	340
	median	183	247
46205	mean	388	365
	median	263	285
Outside Center Township			
46219	mean	157	244
	median	123	204
46220	mean	175	239
	median	110	156

## Supplementary Materials

The following are available online at http://www.mdpi.com/1660-4601/15/7/1531/s1, Figure S1: Principle Component analysis of soil samples from Indianapolis, Indiana, Table S1: All soil geochemical data from Indianapolis, Indiana.
