# Supplementary material for: Mapping the Urban Lead Exposome: A Detailed Analysis of Soil Metal Concentrations at the Household Scale Using Citizen Science
Source: Int J Environ Res Public Health. 2018 Jul 19;15(7):1531. doi: 10.3390/ijerph15071531 (PMC6069257; doi:10.3390/ijerph15071531)
Supplement: Supplementary file 1 [file ijerph-15-01531-s001.pdf]

International Journal of
Environmental Research and Public Health

Supplementary Material

Filippelli et al., IJERPH Urban Exposome

A Principle Component analysis was performed utilizing a subset of 400 soil samples from Indianapolis, that are part of the Healthy Cities Project, that were analyzed by handheld XRF. We found that PC1 accounted for 60% of the variance and PC2 for 12% of the variance. Zinc (Zn), lead (Pb) and arsenic (As) plotted very close to each other on a loading diagram (Fig. 1S) and accounted for a substantial portion of PC2 variance.

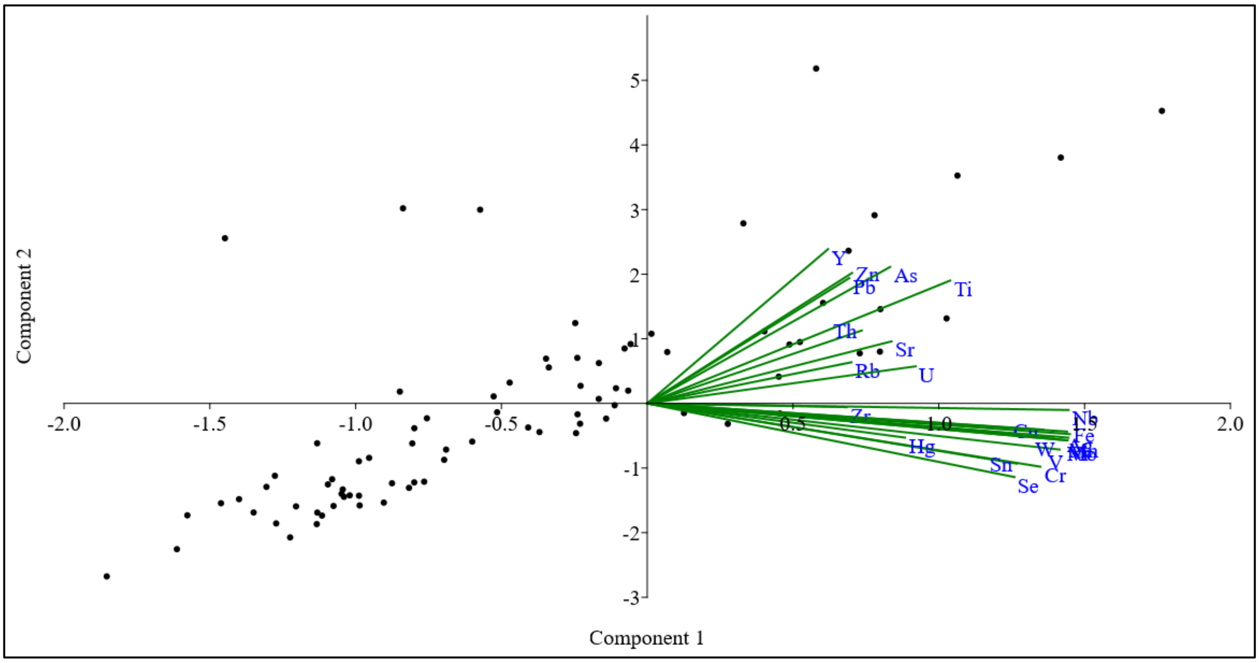

Figure 1S. The variables show a strong positive loading on PC1 and a near split between negative/positive loading on PC2. Note the closeness of the Lead, Zinc, and Arsenic loadings on PC2.

We also determined a host of soil geochemical parameters for the full soil sample set (Table 1S).

Table 1S

Table of all soil geochemical data of each location type in Indianapolis, IN (USA). Rows are multiple elements of the same samples. Soil samples were dried, sieved to 150 microns, and weighed and

ashed in a muffle furnace at 550 degrees Celsius to degrade organic matter. The ashed sample was then transferred to 15 mL HDPE disposable centrifuge tube and digested overnight in 2N HCl at room temperature on a shaker table. After centrifugation, a subsample of the supernatant was diluted (1:100) with Milli-Q water and analyzed on a Perkin Elmer ICP-OES for a suite of metals, specifically lead (Pb), manganese (Mn), barium (Ba), chromium (Cr), copper (Cu), and zinc (Zn). Typical sample reproducibility, calculated from multiple measurements of the same soil ashed, digested, and analyzed was 5% for all elements. Cadmium was also included in the initial analysis, but unacceptably high levels of detection and poor sample reproducibility precluded its effective use in this study.
